# AVPV neurons containing estrogen receptor-beta in adult male rats are influenced by soy isoflavones

**DOI:** 10.1186/1471-2202-8-13

**Published:** 2007-02-01

**Authors:** Lihong Bu, Edwin D Lephart

**Affiliations:** 1 Physiology and Developmental Biology Department and Neuroscience Center Brigham Young University, Provo, UT, USA; 2 Division of Newborn Medicine, Children's Hospital Boston, Harvard Medical School, Boston, MA, 02115, USA

## Abstract

**Background:**

Isoflavones, the most abundant phytoestrogens in soy foods, are structurally similar to 17beta-estradiol. It is known that 17beta-estradiol induces apoptosis in anteroventral periventricular nucleus (AVPV) in rat brain. Also, there is evidence that consumption of soy isoflavones reduces the volume of AVPV in male rats. Therefore, in this study, we examined the influence of dietary soy isoflavones on apoptosis in AVPV of 150 day-old male rats fed either a soy isoflavone-free diet (Phyto-free) or a soy isoflavone-rich diet (Phyto-600).

**Results:**

The occurrence of apoptosis in AVPV was examined by TUNEL staining. The incidence of apoptosis was about 10 times higher in the Phyto-600 group (33.1 ± 1.7%) than in the Phyto-free group (3.6 ± 1.0%). Furthermore, these apoptotic cells were identified as neurons by dual immunofluorescent staining of GFAP and NeuN as markers of astrocytes and neurons, respectively. Then the dopaminergic neurons in AVPV were detected by immunohistochemistry staining of tyrosine hydroxylase (TH). No significant difference in the number of TH neurons was observed between the diet treatment groups. When estrogen receptor (ER) alpha and beta were examined by immunohistochemistry, we observed a 22% reduction of ERbeta-positive cell numbers in AVPV with consumption of soy isoflavones, whereas no significant change in ERalpha-positive cell numbers was detected. Furthermore, almost all the apoptotic cells were ERbeta-immunoreactive (ir), but not ERalpha-ir. Last, subcutaneous injections of equol (a major isoflavone metabolite) that accounts for approximately 70–90% of the total circulating plasma isoflavone levels did not alter the volume of AVPV in adult male rats.

**Conclusion:**

In summary, these findings provide direct evidence that consumption of soy isoflavones, but not the exposure to equol, influences the loss of ERbeta-containing neurons in male AVPV.

## Background

Phytoestrogens are naturally occurring, plant derived, non-steroid molecules that are structurally similar to 17beta-estradiol [1]. Of all the phytoestrogens, soy-derived isoflavones are the most abundant in rodent and human diets, and the most studied in animal and clinical research. Dietary soy isoflavones exist as biologically active aglycones (daidzein and genistein) and biologically inactive glucosides (mainly daidzin and genistin). When consumed, glucosides are hydrolyzed by intestinal glucosidases to daidzein and genistein. Daidzein can be further metabolized to equol, a potent and abundant molecule in rodents [2,3]. The structural similarity between isoflavones and 17beta-estradiol enables these molecules to exert moderate estrogenic or antiestrogenic properties via mammalian estrogen receptors (ER). It is well established that genistein has a greater affinity for ERbeta than ERalpha [4]. Moreover, equol appears to bind ERbeta > ERalpha, in a similar manner to that of genistein [2,5].

The anteroventrol periventricular nucleus (AVPV) is located immediately caudal to the vascular organ of the lamina terminalis and rostral to the suprachiasmatic nucleus [6]. The cells in AVPV project directly to gonadotropin releasing hormone (GnRH)-containing neurons and influence the secretion of luteinizing hormone (LH) in rats [7]. The AVPV is sexually dimorphic, but in contrast to other sexually dimorphic nuclei, the overall volume [8], cell density [9], and the number of dopaminergic neurons [10] are greater in females compared to males in adulthood. These sex differences are regulated by testosterone secreted from the fetal/neonatal testes. There are two surges in circulating testosterone during early development, one occurring around gestation day 18 and the other at approximately 2 hours after birth [11,12]. Within the hypothalamus, testosterone can be converted to 17beta-estradiol by the cytochrome P450 aromatase enzyme, and 17beta-estradiol is thought to be responsible for smaller AVPV characteristics in males [13,14]. This "estrogenic masculinization" process can be manipulated by induction of steroid hormones during the development. Administration of estradiol to neonatal rats is as effective as testosterone in reducing the volume of AVPV [13] by facilitating apoptosis in the developing AVPV [14]. The sexual differentiation of AVPV was thought to be limited to early postnatal period. However, the AVPV characteristics develop as late as 60–80 days after birth [15], and more recent data suggests that they are more plastic than previously thought [2,16].

In light of the estrogenic nature of soy isoflavones and their ability to cross the blood brain barrier [17,18], we have studied the effects of soy isoflavones on characteristics of the AVPV. In two separate studies, we observed a significant decrease in AVPV volume in adult male rats consuming a soy isoflavone-rich diet compared to animals fed a soy isoflavone-free diet [2,16]. However, it is not known whether soy isoflavones act in a similar manner as 17beta-estradiol to alter the volume of AVPV by influencing apoptosis or whether equol contributes to the alteration of AVPV volume. Therefore, in this study we examined the influence of dietary soy isoflavones on apoptosis in adult male rats, by quantifying its incidence, identifying the cell type involved, and exploring its correlation with estrogen receptor subtypes. Finally, the volume of AVPV was quantified in adult male rats after exposure to equol only, a major biologically active isoflavone metabolite.

## Results

### Consumption of soy isoflavones induces apoptosis in AVPV

**Figure 1:**
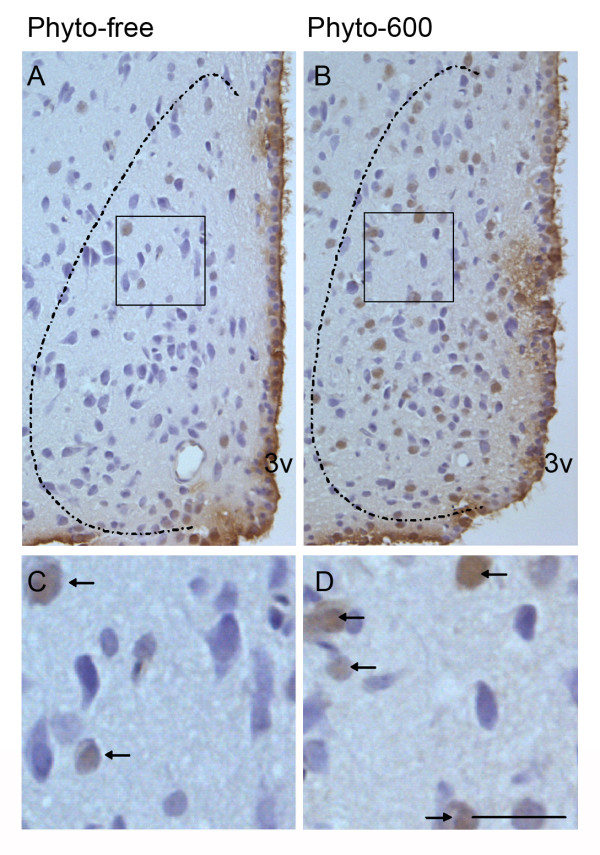
Representative photomicrographs of apoptotic cells labeled by TUNEL staining in AVPV of 150 day-old male Long-Evans rats fed either an isoflavone-free diet (Phyto-free) or an isoflavone-rich diet (Phyto-600) from conception until time tissue collected. Apoptotic cells were visualized with diaminobenzadine (DAB) and seen as brown nuclear staining. Sections were counterstained with hematoxylin (blue). A and C are representative photomicrographs of the Phyto-free AVPV (n = 5). B and D are representative photomicrographs of the Phyto-600 AVPV (n = 5). Across the diet treatments, A corresponds to a similar coronal brain section in B. The AVPV is outlined with dashed lines. Boxed regions in A and B are magnified and shown in C and D, respectively. Significantly more apoptotic cells (arrows) were observed in Phyto-600 AVPV than the Phyto-free group (n = 5; p < 0.001; two-sample student t-test). Bar = 25 μm for all the photomicrographs.

The effects of dietary soy isoflavones on apoptosis in AVPV were examined by TUNEL staining on coronal brain sections, identified as a cell cluster at the rostral level of the third ventricle (Figure 1). The total cell number in AVPV was not significantly altered in the Phyto-600 fed male rats (123.4 ± 3.3) compared to the Phyto-free fed animals (116.0 ± 3.5; Table 1). However, the total apoptotic cell number in the Phyto-600 AVPV (38.4 ± 2.4) was significantly greater than that in the Phyto-free group (4.4 ± 1.2; Table 1, Figure 1). Furthermore, the incidence of apoptosis, calculated as a percentage of the total population of cells, was about 10 times higher in the Phyto-600 group than in the Phyto-free group (Table 1).

**Table 1:** The incidence of apoptosis in the AVPV of adult male Long-Evans rats^a^

Group	Total Cell Number	Apoptotic Cell Number	Incidence of Apoptosis^b^
Phyto-free	126	3	2.4
	125	1	0.8
	113	6	5.3
	133	8	6.0
	120	4	3.3
	***123.4 ± 3.3***	***4.4 ± 1.2^***	***3.6 ± 1.0^***
Phyto-600	111	32	28.8
	112	34	30.1
	108	41	38.0
	124	40	32.3
	125	45	36.0
	***116.0 ± 3.5***	***38.4 ± 2.4***	***33.1 ± 1.7***

^a ^The rats were exposed to either a soy isoflavone-free diet (Phyto-free) or a soy isoflavone-rich diet (Phyto-600) from conception until 150 days of age (n = 5 per diet treatment group).

^b ^Incidence of apoptosis was expressed as the percentage of apoptotic cell number over total cell number in AVPV.

^ Significantly greater apoptotic cell number or incidence of apoptosis in the Phyto-600 AVPV compared to the Phyto-free group (p < 0.05; two sample student t-test).

**Figure 2:**
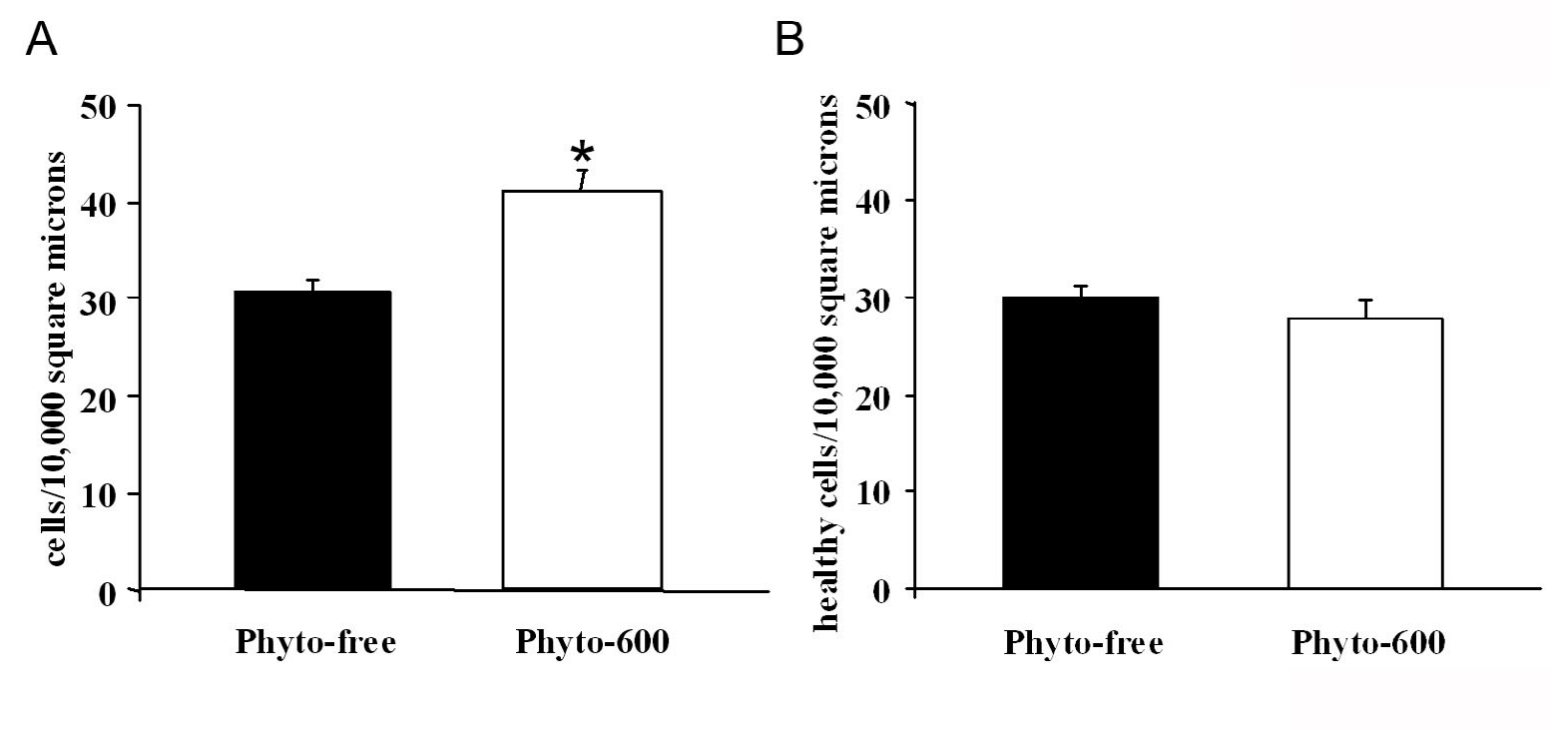
Cell densities in AVPV of 150 day-old male Long-Evans rats fed either an isoflavone-free diet (Phyto-free) or an isoflavone-rich diet (Phyto-600) from conception until time tissue collected. Cell densities in (A) were measured from both apoptotic and non-apoptotic cells in the field of 10,000 μm^2^, while the cell densities in (B) were measured from non-apoptotic cells. Each data point represents mean ± SEM of six measurements (two most condensed fields of three AVPVs out of five animals). * significantly greater cell density in the Phyto-600 fed male rats than the Phyto-free animals (p < 0.001; two-sample student t-test).

When the cell densities within the AVPV were calculated, the rats on the Phyto-600 diet displayed significantly greater cell numbers per unit area than the animals on the Phyto-free diet (Figure 2A). Interestingly, after accounting for the incidence of apoptotic cells, the significant differences between the diet treatment groups for cell densities disappeared (Figure 2B).

### The apoptotic cells induced by soy isoflavones are neurons

**Figure 3:**
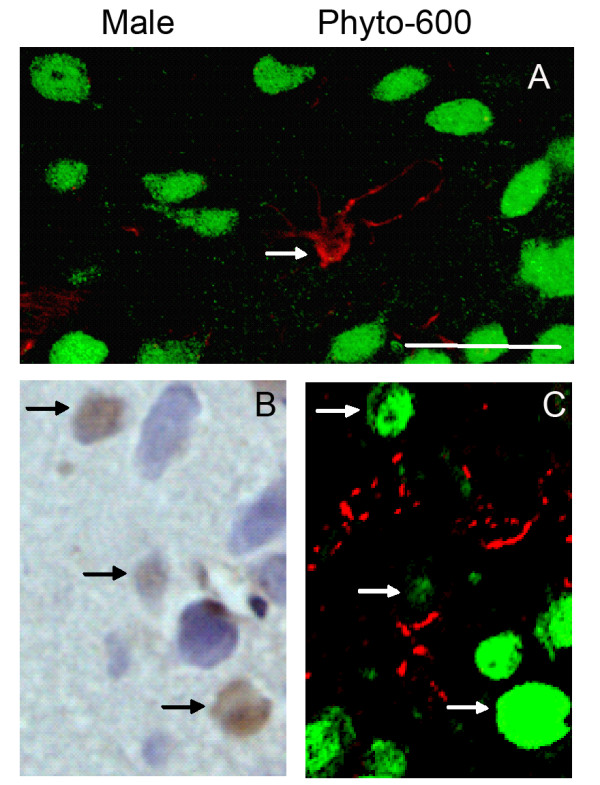
Representative photomicrographs of apoptotic cells (brown nuclear staining), astrocytes (red) and neurons (green) by TUNEL and dual GFAP/NeuN immunofluorescent staining in AVPV of 150 day-old male Long-Evans rats. All the photomicrographs (coronal brain sections) are from animals fed an isoflavone-rich diet (Phyto-600) from conception until time tissue was collected. An astrocyte (red; arrow) is indicated in A, whereas the neurons are displayed in green. B is a representative photomicrograph of TUNEL staining in AVPV (n = 5). C displays dual GFAP/NeuN immunofluoresent stained sections within AVPV, which is 6 μm apart from B. The apoptotic cells (arrows in B) were identified as neurons (arrows in C). Bar = 25 μm for all the photomicrographs.

To determine whether glia or neurons account for the apoptotic cells in AVPV observed previously, we detected the markers for astrocytes (GFAP) and neurons (NeuN) on the adjacent sections of Phyto-600 fed male rats, which were 6 micrometers apart from the TUNEL stained sections. More than 90% of the apoptotic cells were present on both of the consecutive sections. Thus, by locating the apoptotic cells (brown nuclear staining in TUNEL) on the photomicrographs of dual GFAP/NeuN IF staining (neurons were stained green; whereas astrocytes were red), the apoptotic cells were identified as neurons (Figure 3).

### The number of dopaminergic neurons in AVPV was not altered by soy isoflavones

**Figure 4:**
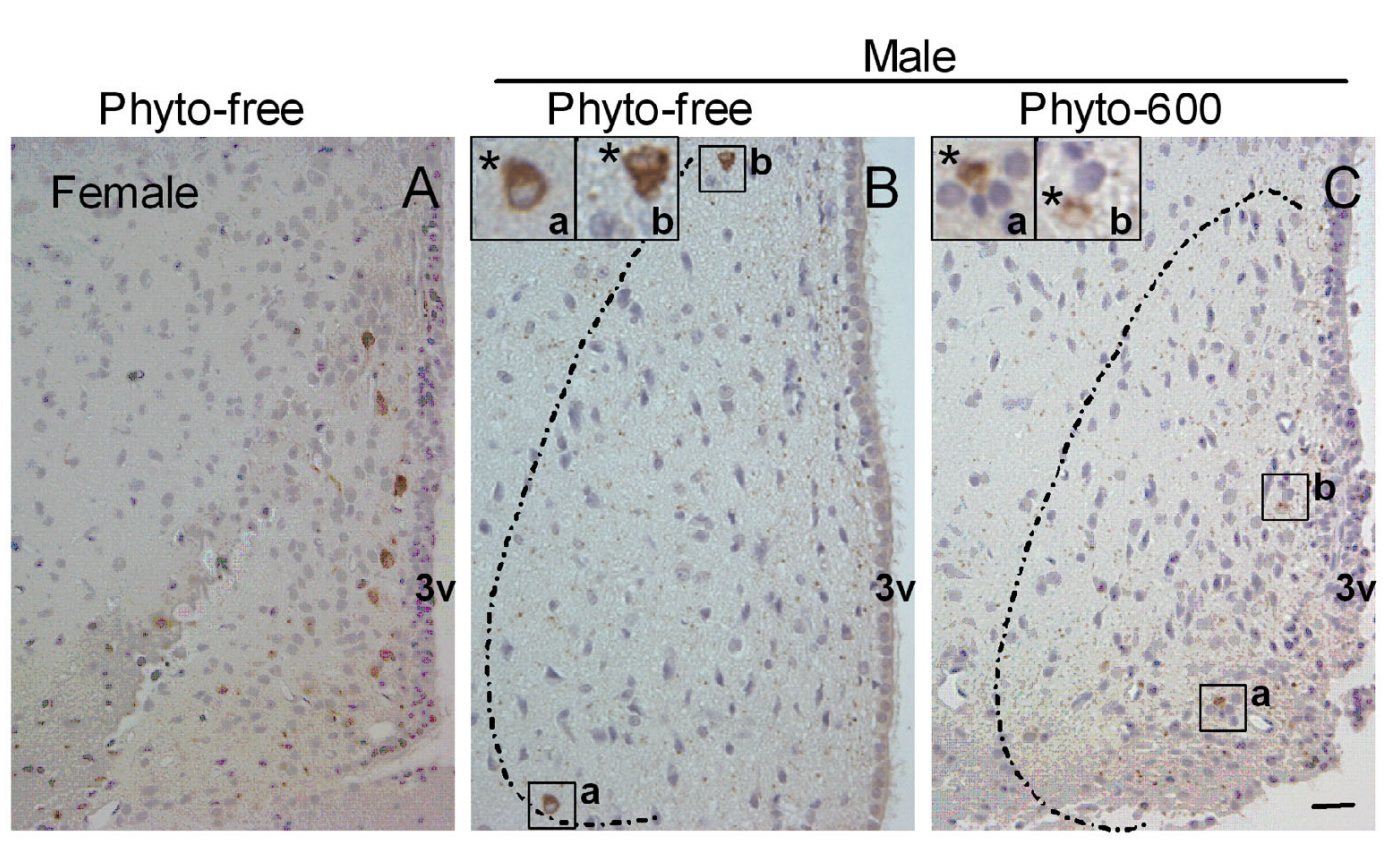
Representative photomicrographs of immunohistochemistry for tyrosine hydroxylase (TH) labeled neurons in AVPV of 150 day-old Long-Evans rats fed either an isoflavone-free diet (Phyto-free) or an isoflavone-rich diet (Phyto-600) from conception until time tissue collected. TH-immunoreactive(ir) cells were visualized with diaminobenzadine (DAB) and seen as brown cytoplasma staining. Sections were counterstained with hematoxylin (blue). Positive control of TH staining is shown in Phyto-free fed female AVPV (A). In males, no significant differences in the number of TH-ir neurons were observed between Phyto-600 (C) and Phyto-free AVPV (B). The TH-ir cells in the boxed regions were indicated (*) at high magnification in the up-left corner of B and C. Bar = 25 μm for all the photomicrographs.

Tyrosine hydroxylase has proven to be a reliable marker for dopaminergic neurons in AVPV even though there are relatively few TH cells in this sexually dimorphic structure. Dopaminergic neurons in AVPV were detected with immunohistochemical staining of TH in 10 day-old male and female and 150 day-old female rats fed the Phyto-free diet and 150 day-old males fed on either Phyto-free or Phyto-600 diet. Independent of diet treatment, we observed significantly more TH neurons in females (approximately 4-fold higher) than in males fed the Phyto-free diet at both ages. However, no significant difference in the sparse number of TH neurons was observed in male rats with long-term exposure to dietary soy isoflavones (Phyto-600) compared to the Phyto-free fed males (Figure 4).

### The apoptotic neurons express ERbeta, but not ERalpha

**Figure 5:**
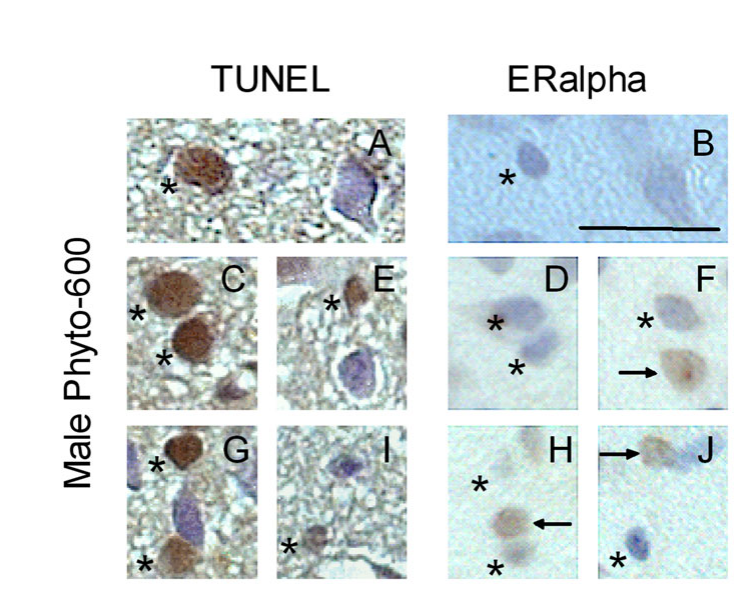
Representative photomicrographs of apoptotic cells (TUNEL) and ERalpha-immunoreactive cells (ERalpha IHC) in AVPV of 150 day-old male Long-Evans rats. All the photomicrographs (coronal brain sections) are from animals fed an isoflavone-rich diet (Phyto-600) from conception until time tissue collected. ERalpha-immunoreactive (ir) cells were visualized with diaminobenzadine (DAB) and seen as brown nuclear staining (arrows in ERalpha). Sections were counterstained with hematoxylin (blue). A, C, E, G and I are representative photomicrographs of TUNEL staining in AVPV (n = 5). B, D, F, H and J are ERalpha IHC stained sections within AVPV, which are 6 μm apart from A, C, E, G, and I, respectively. Apoptotic cells (stars in TUNEL) were not ERalpha-ir (stars in ERalpha). Bar = 25 μm for all the photomicrographs.

**Figure 6:**
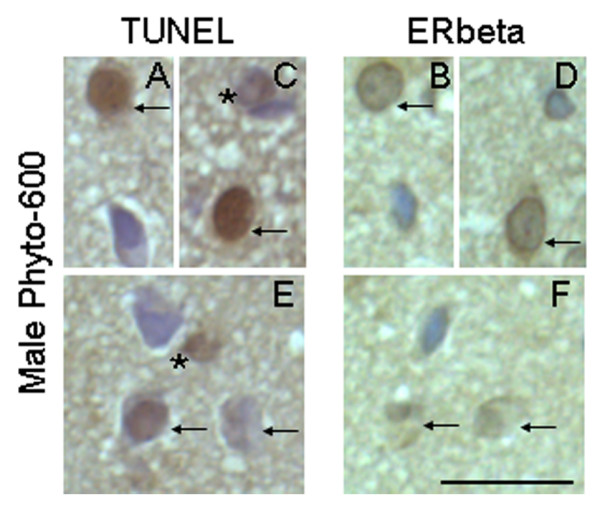
Representative photomicrographs of apoptotic cells (TUNEL) and ERbeta-immunoreactive cells (ERbeta IHC) in AVPV of 150 day-old male Long-Evans rats. All the photomicrographs (coronal brain sections) are from animals fed an isoflavone-rich diet (Phyto-600) from conception until time tissue collected. ERbeta-immunoreactive cells were visualized with diaminobenzadine (DAB) and seen as brown nuclear staining. Sections were counterstained with hematoxylin (blue). A, C and E are representative photomicrographs of TUNEL staining in AVPV (n = 5). B, D and F are ERbeta immunohistochemistry stained sections within AVPV, which are 6 μm apart from A, C and E, respectively. Except a few apoptotic cells (stars in C and E) missing in ERbeta IHC sections, all other apoptotic cells (arrows in TUNEL) were ERbeta-immunoreactive (arrows in ERbeta). Bar = 25 μm for all the photomicrographs.

**Figure 7:**
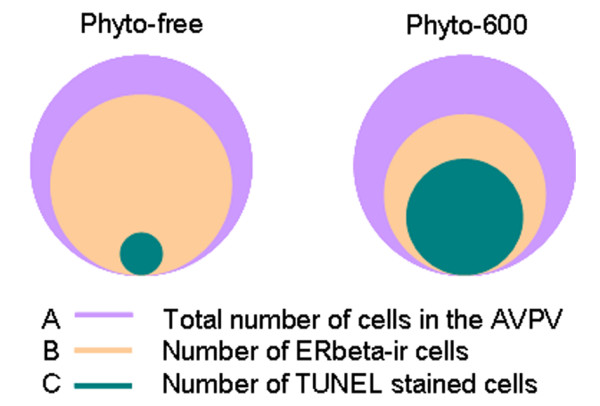
Schematic diagram of the total number of cells relative to the numbers of ERbeta-ir cells and TUNEL stained cells in AVPV of Long-Evans rats fed either an isoflavone-free diet (Phyto-free) or an isoflavone-rich diet (Phyto-600) from conception until time tissue collected. The total number of cells in Phyto-free AVPV is similar to that of Phyto-600 (A). In the Phyto-free group, ERbeta-ir cells represent approximately 67.5% of the total number of cells (B), whereas TUNEL stained cells account for approximately 3.6% of total cells in AVPV (C). In the Phyto-600 group, ERbeta-ir cells represent approximately 52.8% of the total number of cells (B), whereas TUNEL stained cells account for approximately 33.1% of total cells in AVPV (C). Independent of diet treatment, most of the TUNEL stained cells express ERbeta.

**Figure 8:**
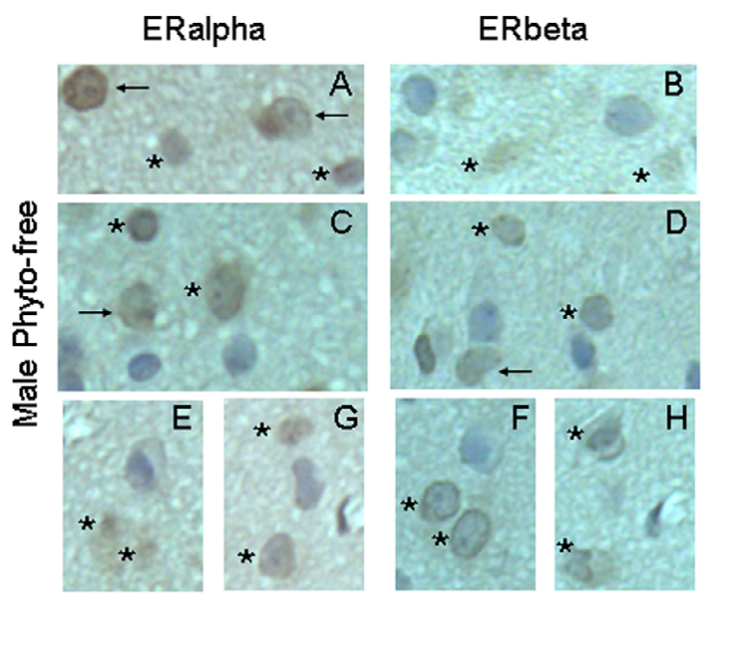
Representative photomicrographs of ERalpha and ERbeta-immunoreactive cells (IHC staining) in AVPV of 150 day-old male Long-Evans rats. All the photomicrographs (coronal brain sections) are from animals fed an isoflavone-free diet (Phyto-free) from conception until time tissue collected. ER-immunoreactive (ir) cells were visualized with diaminobenzadine (DAB) and seen as brown nuclear staining. Sections were counterstained with hematoxylin (blue). A, C, E and G are representative photomicrographs of ERalpha IHC staining in AVPV (n = 2). B, D, F and H are ERbeta IHC stained sections within AVPV, which are 6 μm apart from A, C, E and G, respectively. Some cells are both ERalpha- and ERbeta-ir (stars for ERalpha and ERbeta). The number of cells in the AVPV that express both ERalpha and ERbeta is approximately 20–22% of the total number of cells within this nuclear structure. Some cells are only ERalpha-ir or ERbeta-ir (arrows in ERalpha or ERbeta). Bar = 25 μm for all the photomicrographs.

The expression of ERalpha and ERbeta was detected on coronal brain sections containing AVPV, which were 6 micrometers apart from the TUNEL stained sections. ERalpha and ERbeta immunoreactivity was expressed in cell nuclei of the AVPV (brown nuclear staining; Figure 5 and 6). In AVPV, the number of ERalpha-ir and ERbeta-ir nuclei and total counterstained cell number were counted. The ratio of ERbeta-ir cells in AVPV, expressed as a percentage of the total number of cells, was 52.8 ± 0.6% in the Phyto-600 fed animals. This represents a 22% reduction when compared to the Phyto-free values (67.5 ± 1.0%; data not shown in graph; two-sample student t-test; p < 0.001). No significant differences in the number of ERalpha-ir cells in AVPV were observed between the Phyto-600 group (64.1 ± 1.3%) and Phyto-free group (64.7 ± 1.2%). Furthermore, by locating the apoptotic cells on the sections of ERalpha and ERbeta IHC staining, most the apoptotic cells were ERbeta-ir, but not ERalpha-ir (Figure 5 and 6). In Phyto-600 fed rats, TUNEL-positive cells represent approximately 84% of the ERbeta-ir cells within the AVPV. A schematic diagram displayed in Figure 7 represents the total number of cells in the AVPV relative to the total number of ERbeta-ir cells and TUNEL stained cells. Last, comparing ERalpha and ERbeta IHC sections, we noticed that some cells solely express ERalpha or ERbeta. However, some cells express both ERalpha and ERbeta (Figure 8).

### Exposure to equol (a major isoflavone metabolite) does not alter the volume of AVPV

**Figure 9:**
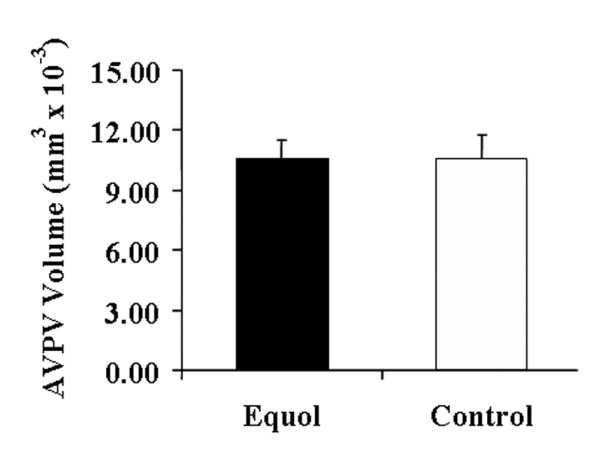
AVPV volumes of adult male Long-Evans rats after equol injection treatment. The volumes were measured and analyzed with Bioquant^®^. No significant alterations were observed between equol-treated compared to the control (DMSO) group (two sample student t-test).

To further test whether equol is the molecule that causes the effects observed above, equol or control (DMSO) vehicle was injected subcutaneously into adult male rats fed the Phyto-free diet. The equol injection treatment was given for 25 consecutive days. The serum circulating equol levels were equivalent to consuming a phytoestrogen-rich soy diet (@ approximately 1,000 ng/ml) [2]. Interestingly, there were no significant alterations in AVPV volumetric morphometric parameters with the exposure to equol (Figure 9). So, exposure solely to equol is not sufficient to alter the hormone-sensitive hypothalamic volume (AVPV) in male rats during adulthood which is opposite to the consumption of soy (phytoestrogens) via dietary routes for an equivalent exposure interval previously observed by our laboratory [2,16].

## Discussion

This study directly demonstrates that long-term exposure to dietary soy isoflavones induces neuronal apoptosis in the AVPV of adult male rats. Additionally, the present data suggests that the influence of soy isoflavones on neuronal apoptosis is not correlated to ERalpha but dependent on ERbeta. However, exposure to equol is not sufficient to alter the volume of AVPV.

Neurons in the AVPV are born between gestation day 13 and gestation day 18 [19]. Neither neurogenesis [20] nor neuronal migration [21] has been reported to play a role in the development of sexual dimorphism in the preoptic area. However, it has been demonstrated that apoptosis is the major way that steroid hormones alter neuronal numbers in sexually dimorphic regions during critical periods of development [14,22]. This critical period for sexual differentiation was revised to be longer than the perinatal and first week postnatal window [15], and the volume of the sexually dimorphic nuclei can still be changed by exposure to estrogenic molecules in adults [2,16]. Therefore, it is novel, but not surprising, to observe significantly higher incidence of apoptosis in the AVPV of adult male rats consuming soy isoflavones, via their estrogenic actions. Generally, TUNEL is a well-accepted technique to detect apoptotic cells by visualization of DNA fragmentation [23]. Cells with DNA damage may also be identified by TUNEL staining. Thus, the TUNEL-positive cells in this study include apoptotic cells and possibly cells in the process of DNA repair [24]. Furthermore, these TUNEL-positive cells were identified as neurons. This is the first direct evidence of soy isoflavones inducing neuronal apoptosis in AVPV *in vivo*. Additionally, taking the increased cellular density and the similar total cell number in the Phyto-600 AVPV sections together, these data implied smaller AVPV volumes in the Phyto-600 group vs. the Phyto-free group, which is consistent with our previous findings [2,16]. Implications to human health and reproduction are unknown, especially since reproductive capacity of Asian populations appears to be unaffected by consumption of soy foods [25].

It is known that the number of dopaminergic neurons in AVPV is 3–4 times higher in adult female rats than in males [26]. Although TH neurons only contribute approximately 1% of the total number of cells in male AVPV (personal communication with E.M. Waters by permission), no significant difference in the number of TH neurons was observed in adult male rats between the treatment groups. There is evidence that circulating gonadal steroids appear to downregulate TH expression in both male and female AVPVs [27]. The development of sexually dimorphic TH neurons is not affected by the overexpression of Bcl-2 [28] or deletion of Bax [29], but is dependent on ERalpha [30]. The lack of changes in the number of TH neurons in the male (present study) and female (our unpublished data) rats with long-term exposure to isoflavones (Phyto-600) suggests that the neuronal apoptosis may be independent of ERalpha. Furthermore, the incidence of apoptosis was approximately 33% and the ratio of ERalpha-ir cells over the total cells in AVPV was approximately 64% in the Phyto-600 group. Moreover, in this case the apoptotic cells were not ERalpha-ir. This suggests that ERalpha is not involved in the apoptosis by consumption of soy isoflavones.

The co-localization of ERbeta and DNA fragmentation implies ERbeta may mediate the neuronal apoptosis induced by soy isoflavones in male AVPV. This is consistent with several lines of evidence. First, both ERalpha and ERbeta have been reported to be expressed in the AVPV [31-33]. The expression of ERbeta is sexually dimorphic in rodents. In mice, females a significantly larger number of ERbeta-positive cells were positioned in the medial portion of the AVPV close to third ventricle; in males the distribution of ERbeta-positive cells were dispersed throughout the AVPV [32]. The apoptotic cells observed in the Phyto-600 AVPVs did not show a specific pattern, in other words, similar to the expression of ERbeta in male AVPV. Second, it has been reported that estrogen-regulated developmental neuronal apoptosis is determined by ER subtype: ERalpha has a neuroprotective effect, while ERbeta medicates the induction of apoptosis in neuronal cells [34]. Third, dopaminergic neurons express ERbeta in the female AVPV, but not in males [32]. This partially favors that ERbeta-expressing dopaminergic neurons go through apoptosis in males in the presence of testosterone/estrogen, but not in females. On the contrary, in females these cells are thought to survive. Fourth, ERalpha and ERbeta are coexpressed in some cells in AVPV [35]. When coexpressed, ERalpha and ERbeta form functional heterodimers [36]. Even though the biological roles of ERalpha/beta heterodimers in the presence of each respective homodimer are unknown, ERbeta exhibits an inhibitory action on ERalpha-mediated gene expression and in many instances opposes the actions of ERalpha [36]. Additionally, we observed more ERbeta-ir cells than apoptotic cells in AVPV. Hence, we speculate neurons that coexpressed ERalpha and ERbeta may escape apoptosis, whereas those expressing only ERbeta are sensitive to the estrogenic signal and showed DNA fragmentation. Finally, although isoflavones are less potent than 17beta-estradiol, the plasma concentrations of genistein (117 ± 5 ng/ml) and equol (1363 ± 59 ng/ml) in the Phyto-600 fed male rats are much greater than that of 17beta-estradiol (1–5 pg/ml) [25]. The average total isoflavone content within the hypothalamus is more than 3-fold higher for Phyto-600 fed males (134 ng/g) vs. Phyto-free fed males (40 ng/g) [17]. Furthermore, isoflavones possess a higher affinity for ERbeta than ERalpha [4]. So the influence of isoflavones is sufficient to cause a variety of biological effects via estrogen receptors, especially ERbeta. We speculate that genistein or other isoflavone molecule(s) may be responsible for the increased neuronal apoptosis observed in this study.

It is intriguing to consider the following hypothesis as to how dietary soy isoflavones modulate AVPV cell and volume characteristics via ERbeta. First, it is well established that estrogens decrease AVPV volumes during pre- and postnatal development [37]. Second, it is also well established that the aromatase cytochrome P450 enzyme (that converts androgens to estrogens) is present in neuronal hypothalamic regions and that aromatase mRNA expression and aromatase enzymatic levels decline with increasing postnatal age, especially after puberty, compared to the prenatal developmental interval [38]. Thus, even though there is abundant steroid substrate from the testes (i.e. testosterone) during adulthood, the levels of hypothalamic aromatase decline more than 50-fold compared to prenatal levels. This dramatically decreases the local biosynthesis of estrogens. In this way, consumption of an isoflavone-rich diet (Phyto-600) greatly increases the concentration of estrogen-like molecules in the circulation and in the hypothalamus (see above). Therefore, this would suggest that there are very low levels of endogenous estrogens being formed within hypothalamic regions, while very high circulating estrogen-like isoflavone molecules having the ability to bind ERbeta account for the increase in apoptosis in the AVPV of Phyto-600 fed males, versus very little apoptosis was as seen in the AVPV of Phyto-free fed males. Compared with the "estrogenic masculinization" during male AVPV development, the "dietary phytoestrogenic masculinization" is likely to be a mechanism underlying the loss of ERbeta-containing neurons we observed in these adult male rats. Even though it is not certain that the increased cell death in adult male AVPV is beneficial; it could be speculated that it is not detrimental. The influence of dietary isoflavones on apoptosis was studied in the present study at 150 days of age when the characteristics of AVPV have been fully developed while the volume of AVPV was reported to be affected by dietary treatment. Further research at different developmental stages is essential to explore how isoflavones influence neuronal apoptosis.

Finally, plenty of information in the literature shows that conventional research and development approaches fail to fully isolate or identify chemicals for synthesis of analogues from well-known Chinese medicinal plants [39]. It is not surprising that equol injection, with equivalent exposure level and interval to dietary consumption described previously, did not alter hormone-sensitive hypothalamic volumes in rats during adulthood. This indicates that multiple factors or a combination with genistein may be required to alter brain structures in the sensitive rat model.

## Conclusion

The present experiments demonstrate that the consumption of soy isoflavones 1) induces neuronal apoptosis and/or DNA fragmentation in the AVPV of adult male rats, 2) the number of ERalpha-dependent dopaminergic neurons is not altered by the diet treatments, and 3) almost all the apoptotic cells are ERbeta-ir, but not ERalpha-ir. However, exposure to equol does not alter AVPV volume. In summary, these findings provide direct evidence that consumption of soy isoflavones influences loss of ERbeta-containing neurons in male AVPV.

Since soy dietary content is usually not considered as an experimental variable, future research designs should take into account this potential important and pervasive hormonal factor.

## Methods

### Dietary treatment

Long-Evans rats (12 males and 16 females) were purchased from Charles River Laboratories (Wilmington, MA, USA) at 50 days of age for breeding. These animals were caged individually and housed in the Brigham Young University Bio-Ag vivarium and maintained on an 11-hour dark and 13-hour light schedule (lights on 0600–1900). The use of animals and the methods of this study were approved by the Institute of Animal Care and Use Committee (IACUC) at Brigham Young University (BYU).

Upon arrival all animals were allowed ad libitum access to water and either a commercially available diet with high phytoestrogen levels (Harlan Teklad Rodent Diet 8604, Madison, WI, USA) containing approximately 600 ppm of soy isoflavones (referred to hereafter as the Phyto-600 diet), or a custom diet (Ziegler Bros., Gardner, PA, USA) containing approximately 10–15 ppm of soy isoflavones (referred to hereafter as the Phyto-free diet) [40]. The content and nutrient composition of these diets is described in detail elsewhere [40]. The diets were balanced and matched for equivalent percentage content of protein, carbohydrate, fat, amino acids, vitamins and minerals, etc [40]. Circulating phytoestrogen serum levels from rats maintained on these diets have been reported previously by our laboratory using GC/MS analysis [2,16,18,40]. The animals were time mated within their respective diets so that the offspring of these pairings would be exposed solely to either the Phyto-600 or Phyto-free diet.

### Brain sample preparation

At approximately 150 days of age, the male offspring (n = 5) by diet treatment were deeply anesthetized with a mixture of ketamine/acepromazine (75/2.5 mg/kg IP) and transcardially perfused with isotonic saline and then 10% buffered formalin. The whole brain was immediately removed from the skull and stored in 10% buffered formalin for 14 days and 10% sucrose for one week before being embedded in paraffin. Coronal brain sections were prepared at 6 micrometers with a microtome. The AVPV was located by using landmarks such as the anterior commissure and third ventricle. Then sections on the exact same plane from different animals were processed for further comparisons between animals described below.

### TUNEL staining

To detect apoptosis in AVPV, NeuroTACSTM II (a reagent kit for in situ detection of apoptosis in neural tissue; Cat # 4823-30-K) was purchased from Trevigen, Inc. (Gaithersburg, MD, USA). NeuroTACS II utilizes terminal deoxynucleotidyl transferase (TdT) to incorporate biotinylated nucleotides at the sites of DNA breaks which are characteristic of apoptosis. The deparaffinized slides were stained according to the manufacturer's direction. Briefly, brain sections were rehydrated in ethanols and permeablized. Endogenous peroxidase was inactivated by 3% hydrogen peroxide. The sections were incubated in a humidity chamber with labeling reaction at 37°C for 1 h, then with streptavidin HRP for 15 min at room temperature. Next they were developed with diaminobenzidine (DAB) and counterstained with hematoxylin. After dehydration, the slides were coversliped with Permount (Cat # 26905, Richard-Allan Scientific, Kalamazoo, MI, USA).

The samples were viewed with an Olympus BX61 microscope using 40× objectives. TUNEL-positive apoptotic cells exhibited a brown nuclear staining. The total number of counterstained cells in AVPV in a single section was counted (n = 5). Then, the total number of apoptotic cells in AVPV on the same section was recorded. Additionally, the total cumulative incidence of apoptosis was calculated as a percentage of the total population of cells. Then the cellular density of the AVPV was calculated by counting the cell number in 2 most condensed fields of 100 × 100 μm within AVPV in each section and expressed as number of cells per 10,000 μm^2^. For each group, a total of 6 measurements on cellular density were performed.

### Dual immunofluorescent (IF) staining of GFAP and NeuN

After TUNEL staining, the adjacent sections were stained with dual immunofluorescent staining of GFAP and NeuN (the markers for astrocytes and neurons, respectively). GFAP (Glial Fibrillary Acidic Protein), the main constituent of intermediate filament of astrocytes, is found in the cytoplasm and appendages. NeuN (NEUronal Nuclei) is found only in neurons. The deparaffinized and rehydrated sections were microwaved in 10 mM Sodium Citrate buffer (antigen retrieval) at full power (900 W) for 1 min, followed by 9 min at half power and 20 min to cool down. After being blocked in 3% goat serum in PBS for 1 h, the sections were incubated in a humidity chamber at 4°C overnight with the primary antibodies, a rabbit anti-GFAP (1:1000, Cat # AB5804, Chemicon, Temecula, CA, USA) and a mouse anti-NeuN (1:100, Cat # MAB377, Chemicon, Temecula, CA, USA). The sections were then rinsed three times in phosphate buffered saline (PBS) for 5 min each and incubated in a humidity chamber for 1 h at room temperature with secondary antibodies, a far-red-fluorescent Alexa Fluor 633 dye labeled goat anti-rabbit IgG (1:200; Cat # A-21072, Molecular Probes, Eugene, OR, USA) and a bright green-fluorescent Alexa Fluor 488 dye labeled goat anti-mouse IgG (1:200; Cat # A-11070, Molecular Probes, Eugene, OR, USA). Then, the samples were rinsed in PBS three times for 5 min each and mounted with Fluoromount-G (Cat # 0100-01, Southern Biotechnology Associates, Inc., Birmingham, AL, USA). Dual-immunofluorescent specimens were analyzed at high power (60× objective lens; oil) with an Olympus FluoView FV300 confocal microscope (Minneapolis, MN, USA) using Blue Argon (488 nm) laser and Red Helium Neon (633 nm) laser.

### Immunohistochemical (IHC) staining of tyrosine hydroxylase (TH)

As described above for immunofluorescent staining, the antigen was retrieved by incubation in 10 mM sodium citrate buffer and endogenous peroxidase was inactivated by 3% hydrogen peroxide. Then the samples were blocked in 3% goat serum in PBS and incubated at 4°C overnight in primary antibody solution of goat anti-TH (1:500, Cat # AB152, Chemicon, Temecula, CA, USA), which was localized with a biotinylated goat anti-rabbit IgG (1:500, Cat # AP132P, Chemicon, Temecula, CA, USA; 1 h at room temperature). Staining (DAB), counterstaining (hematoxylin), mounting and viewing was the same as described for TUNEL staining.

### Immunohistochemical (IHC) staining of estrogen receptor (ER) alpha and beta

As described above for TUNEL and TH IHC staining, the sections were deparaffinized, rehydrated and permeablized, followed by the antigen retrieval in sodium citrate buffer and inactivation of endogenous peroxidase in 3% hydrogen peroxide. After being blocked in 3% goat serum in PBS for 1 h at room temperature, the sections were incubated in a humidity chamber at 4°C overnight with rabbit anti-ERalpha (1:1000, Cat # 06-935, Upstate, Lake Placid, NY, USA) or rabbit anti-ERbeta (1:100, 10 μg/ml, Cat # 06-629, Upstate, Lake Placid, NY, USA; these antibodies have been employed previously with validated methods [41,42]. Immunoparticipate was visualized by an ABC Elite kit and DAB methods (Cat # PK-6101 and Cat # SK-4100, respectively, Vector Laboratories, Burlingame, CA, USA). Counterstaining (hematoxylin), mounting and viewing was the same as described for TUNEL staining.

ER-positive apoptotic cells exhibited a brown nuclear staining. The total number of counterstained cells in AVPV in a single section was counted (n = 5). Then, the total number of ER-positive cells in AVPV on the same section was recorded. Finally, the percentage of ER-positive cells of total cells in AVPV was calculated for each animal. Negative controls for all labeling studies were carried out by omitting TdT (TUNEL) or the primary antibodies.

### Equol injection treatment

Male Long-Evans rats at 50 days of age were placed on the Phyto-free diet. At 150 days of age the rats were matched by body weight and then divided into control and equol treatment groups (n = 4). At 190 days, the rats received daily s.c. (0.1 cc) injections of control vehicle (DMSO) or equol at approx. 2.5 mg/Kg for 25 consecutive days. At 215 days of age the animals were sacrificed, blood was collected for equol levels; the brains processed via standard staining and analyzed via Bioquant^® ^for morphometric AVPV parameters by treatments [2,16]. The serum equol levels were equivalent to consuming Phyto-600 diet.

### Statistical analysis

All the data were expressed as Mean ± SEM and were tested by 2-sample Student T-test in Minitab. Values were considered significantly different if p < 0.05.

## Competing interests

The author(s) declare that they have no competing interests.

## Authors' contributions

LB and EDL were involved in the experimental design, conducting of the research protocol, tissue and data collection and writing of the manuscript. All authors read and approved the final manuscript.
